# Binding Mode and Selectivity of Steroids towards Glucose-6-phosphate Dehydrogenase from the Pathogen *Trypanosoma cruzi*

**DOI:** 10.3390/molecules21030368

**Published:** 2016-03-17

**Authors:** Cecilia Ortiz, Francesca Moraca, Andrea Medeiros, Maurizio Botta, Niall Hamilton, Marcelo A. Comini

**Affiliations:** 1 Redox Biology of Trypanosomes, Institut Pasteur de Montevideo, Mataojo 2020, Montevideo 11400, Uruguay; cortiz@pasteur.edu.uy (C.O.); amedeiros@pasteur.edu.uy (A.M.); 2 Dipartimento di Biotecnologie, Chimica e Farmacia, Università degli Studi di Siena, Via Aldo Moro 2, Siena 53100, Italy; francesca.moraca@gmail.com (F.M.); botta.maurizio@gmail.com (M.B.); 3 Sbarro Institute for Cancer Research and Molecular Medicine, Center for Biotechnology, College of Science and Technology, Temple University, BioLife Science Building, Suite 333, 1900 N 12th Street, Philadelphia, PA 19122, USA; 4 Departamento de Bioquímica, Facultad de Medicina, Universidad de la República, Av. Gral. Flores 2125, Montevideo 11800, Uruguay; 5 Drug Discovery Unit, Cancer Research, UK Manchester Institute, Wilmslow Road, Manchester M204BX, UK; Niall.Hamilton@cruk.manchester.ac.uk

**Keywords:** epiandrosterone, pentose phosphate pathway, Chagas disease, *Leishmania*, inhibition by steroids, structure activity relationship

## Abstract

Glucose-6-phosphate dehydrogenase (G6PDH) plays a housekeeping role in cell metabolism by generating reducing power (NADPH) and fueling the production of nucleotide precursors (ribose-5-phosphate). Based on its indispensability for pathogenic parasites from the genus *Trypanosoma*, G6PDH is considered a drug target candidate. Several steroid-like scaffolds were previously reported to target the activity of G6PDH. Epiandrosterone (EA) is an uncompetitive inhibitor of trypanosomal G6PDH for which its binding site to the enzyme remains unknown. Molecular simulation studies with the structure of *Trypanosoma cruzi* G6PDH revealed that EA binds in a pocket close to the G6P binding-site and protrudes into the active site blocking the interaction between substrates and hence catalysis. Site directed mutagenesis revealed the important steroid-stabilizing effect of residues (L80, K83 and K84) located on helix α-1 of *T. cruzi* G6PDH. The higher affinity and potency of 16α-Br EA by *T. cruzi* G6PDH is explained by the formation of a halogen bond with the hydrogen from the terminal amide of the NADP+-nicotinamide. At variance with the human enzyme, the inclusion of a 21-hydroxypregnane-20-one moiety to a 3β-substituted steroid is detrimental for *T. cruzi* G6PDH inhibition. The species-specificity of certain steroid derivatives towards the parasite G6PDH and the corresponding biochemically validated binding models disclosed in this work may prove valuable for the development of selective inhibitors against the pathogen’s enzyme.

## 1. Introduction

The genera *Trypanosoma* and *Leishmania* encompass parasite species causing severe and, if left untreated, fatal diseases in animals and humans [1]. So far, vaccine development against trypanosomiasis and leishmaniasis appears unattainable due to the capacity of the parasites to evade the host immune response. Thus, the discovery of new chemotherapeutic approaches remains the most reliable strategy to combat these pathogens [2].

Several studies indicate that the glucose-based metabolism of trypanosomatids offers the possibility for selective pharmacological intervention, because of the biochemical and structural differences of some of its components with their human counterparts [3,4]. Like most organisms, trypanosomatids metabolize glucose via the glycolytic or Embden-Meyerhof pathway, and the pentose phosphate pathway (PPP) [5,6]. The first reaction of the PPP is catalyzed by glucose-6-phosphate dehydrogenase (G6PDH) that oxidizes G6P to 6-phosphogluconolactone with concomitant reduction of NADP^+^ to NADPH, which fuels several biosynthetic reactions and antioxidant systems. In *T. cruzi*, G6PDH appears to play an important role during infection since its content was significantly higher in the infective stages and its activity was increased about 45-fold in infective trypomastigotes challenged with H_2_O_2_ [7]. Epigenetic interference with G6PDH expression in the bloodstream form of African trypanosomes demonstrated that the enzyme is essential for parasite survival [8].

The steroids dehydroepiandrosterone (DHEA) and epiandrosterone (EA), first discovered as inhibitors of the human G6PDH [9], were later reported to target the trypanosomal but not the leishmanial enzyme [10]. In agreement with these results, both compounds were cytotoxic towards *T. brucei* and *T. cruzi* but not against *L. mexicana* [8,10,11]. Moreover, African trypanosomes deficient in the endogenous G6PDH but expressing an ectopic copy of *L. mexicana* G6PDH were resistant to EA [11], thus confirming the refractoriness of the leishmanial enzyme to steroids. *In vitro*, G6PDH from *T. brucei* was about 6-fold more sensitive towards inhibition by steroids (Ki value of 1.7 and 0.5 μM for DHEA and EA, respectively) [8] than the *T. cruzi* (Ki value of 2.6–25 μM and 1–5.6 μM for DHEA and EA, respectively) [10,12] and the human homologue (Ki value of 6–9 μM and 3 μM for DHEA and EA) [13]. Recently, a drug screening campaign with a large commercial library of wide chemical diversity led to the identification of new uncompetitive inhibitors of *T. cruzi* G6PDH (*Tc*G6PDH) that belonged to the thienopyrimidine and quinazoline families [12].

In an effort to obtain information about the binding of steroidal inhibitors to G6PDH, Zhao *et al.* performed *in silico* studies with DHEA and derivatives using the structure of human G6PDH with bound G6P as molecular target [14]. The work proposes that H-bonds and non-polar interactions with residues close to the enzyme active site stabilize and orient the inhibitor in the binding site. However, the binding model is not compatible with the uncompetitive inhibition mechanism described for steroids against G6PDH from different species [8,10,13], since the inhibitors partially occupy the nicotinamide nucleotide binding region.

*Tc*G6PDH in its apo- and substrate-bound form has been recently crystallized by our group and the corresponding structures solved (PDB 4E9I and PDB 4EM5, respectively) [15]. Here we exploit the structural information available for *Tc*G6PDH to address the binding mode of steroids to the enzyme. Structural models of steroids bound to *Tc*G6PDH were generated *in silico* and subjected to validation with enzyme point mutants and SAR analysis with different novel steroids [16]. Our study discloses the region and residues of *Tc*G6PDH involved in steroid binding and paves the way for the further challenging design of derivatives targeting selectively the pathogen’s enzyme.

## 2. Results and Discussion

### 2.1. EA Binding to the T. cruzi G6PDH-G6P-NADP^+^ Ternary Complex

The crystal structure of apo- (PDB 4E9I) and G6P-bound *Tc*G6PDH (PDB 4EM5) have recently been obtained [15] and a full structural characterization will be reported elsewhere. *Tc*G6PDH is a tetrameric protein with each subunit composed of an N-terminal domain with a typical Rossmann-fold involved in NADP^+^ binding and a C-terminal domain that contains the G6P binding site and residues engaged in subunit interactions [15]. The catalytic site of G6PDH localizes at the interface between the N- and C-terminal domains and far from regions participating in protein dimerization and tetramerization [17,18,19].

**Figure 1:**
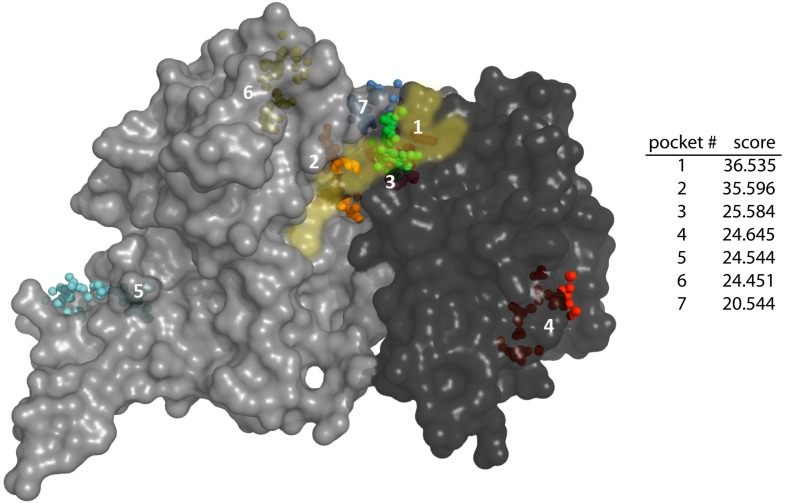
Pocket detection on G6P-*Tc*G6PDH using Fpocket algorithm. Top-7 pockets on the holo-*Tc*G6PDH (PDB 4EM5). *Tc*G6PDH surface representation in grey color (dark and light grey denote the N-terminal and C-terminal protein domains, respectively) with substrates binding sites highlighted in yellow transparent. The alpha sphere centres corresponding to the different cavities are depicted as small colored spheres and the pockets labeled according to their score ranking shown in the table on the right.

As a first approach to identify potential binding sites for steroids in *Tc*G6PDH, we have used the Fpocket algorithm that is based on Voronoi tessellation to detect protein cavities geometrically suitable and accessible to ligands [20]. Inspection of the coordinates for G6P-bound *Tc*G6PDH (PDB 4EM5) with Fpocket revealed a total of 21 pockets with seven of them presenting a score >20, indicating that they can be investigated as putative binding sites for inhibitors. Most of these cavities correspond to regions encompassing or close to the substrates binding sites (*i.e.*, pockets 1, 2, 3, 6 and 7; Figure 1).

Among them, the pockets presenting the highest score localized next to the NADP^+^ binding site (pocket 1, score = 36.535), between the G6P and NADP^+^ binding sites (pocket 2, score = 35.596) or within the NADP^+^ binding site (pocket 3, score = 25.584). In contrast to pockets 2 and 3, and despite its high score, cavity 1 appears more buried in the protein structure and is unlikely to be accessible to the bulky structure of steroids. Nonetheless, ligand binding to any of these sites is expected to influence substrate binding and/or catalysis. Two additional pockets (4 and 5) with relatively good score (score = 24.6) localize in regions far from the active site (Figure 1). Pocket 4 is located on a solvent-exposed area of the Rossmann fold domain, distal from the active site and shaped by residues located in loops from structural elements contributing to NADP^+^ binding. Pocket 5 is located in a non-structured region of the C-terminal domain that is involved in contacts between protein subunits. Because of the likely dynamic nature of the elements involved in forming pockets 4 and 5, the lack of evidence demonstrating steroids affect NADP^+^-binding ([8,9,10,13] and this work) and the oligomeric structure of G6PDH (not shown), these sites were ruled out as potential cavities for EA binding. Instead, the results obtained for the top-three pockets surrounding the catalytic site guided the docking and molecular dynamics studies.

Taking into account that steroids are uncompetitive inhibitors of G6PDH [8,9,10,13] and such inhibition mechanism implies the binding of the inhibitor to the enzyme-substrate(s) complex, a model of the *Tc*G6PDH ternary complex was first generated. Analysis of the crystal structure of apo- or G6P-bound *Tc*G6PDH shows that non-structured elements from the Rossmann-fold, which are critical for anchoring the adenine-phosphate nucleotide moiety of NADP^+^, present conformations not compatible with co-substrate binding (Figure S1). Thus, first NADP^+^ was docked to the structure of *Tc*G6PDH with bound G6P (PDB 4EM5) using the induced fit algorithm of the Schrödinger suite. Nineteen models were obtained and those showing an orientation of substrates consistent with the catalytic mechanism of G6PDH [21,22] were chosen for further docking of EA and analogues thereof. Docking of EA in the newly generated *Tc*G6PDH/G6P/NADP^+^ complex has been performed by means of Glide, using the Standard Precision (SP) scoring function [23]. The resulting binding mode of EA, with the steroid occupying a cavity between G6P and NADP^+^ (*i.e.*, pocket 2 according to Fpocket detection) is consistent with its uncompetitive mode of inhibition. In particular, EA shows an H-bond between its hydroxyl group and R408, being mostly stabilized by hydrophobic contacts with both the protein and G6P. The stability of the *Tc*G6PDH/G6P/NADP^+^/EA complex was checked by means of 80 ns MD simulation. Surprisingly, during the simulation G6P loses the H-bonds with the two catalytic His residues (H247 and H309) [18,19] and, consequently, is displaced from the catalytic site, which precludes electron exchange between G6P and NADP^+^.

In the molecular dynamic model, G6P shows H-bonds with R408 (total occupancy = 99.4%), K251 (total occupancy = 60.9%), H247 (total occupancy = 12.2%) and H309 (total occupancy = 0.19%). On the other hand, EA shows H-bonds only with E216 with a significant occupancy over the 80 ns MD simulation (total occupancy = 31.5%). At the end of the 80 ns, EA loses its contacts with the protein exiting its binding site (Figure S2). The NADP^+^ cofactor, remains in its binding site over the whole simulation. Indeed, as can be seen from the RMSF plots (Figure S3), residues from the catalytic site (D246, H247 and H309), and other residues involved in stabilizing G6P- (R408) or EA-binding (L80, K83 and K84) to the protein, present considerable fluctuations, while residues from the NADP^+^ binding site remain more stable throughout the simulation. Taken together, the MD simulation results support a steric effect of the inhibitor in the proximity of the catalytic site that hampers catalysis.

**Figure 2:**
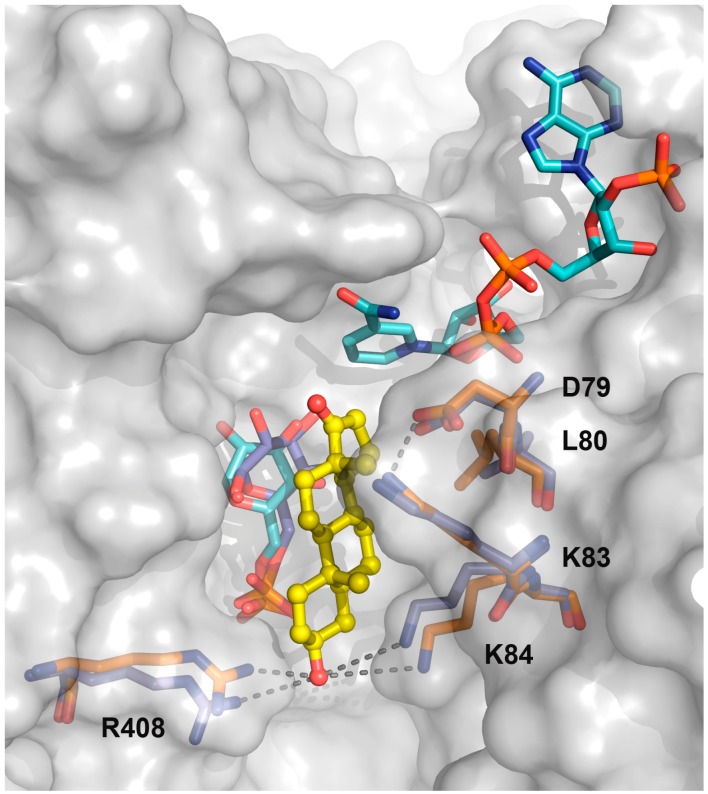
Binding mode of EA to the ternary complex of *Tc*G6PDH. Superimposition of the crystal structure of *Tc*G6PDH with bound G6P (PDB 4EM5, residues shown as violet sticks) with the molecular model of the corresponding energy minimized quaternary complex of the enzyme (protein surface is colored in grey, residues and substrates are shown as bright orange and cyan sticks, respectively, and EA is depicted as yellow balls and sticks). The dashed lines denote the interactions described in the text. The RMSD value for the backbone of both structures is 0.719 Å.

Considering that the success rate of Fpocket to identify sites of protein-ligand interaction has been reported to be over 90% for the best three pockets [20], the docking grid of EA was centered on G6P with an inner and outer box of 20 and 40 cubic Å, respectively, which comprises the area of the top-3 ligand-binding sites previously identified with Fpocket. The stability of the G6PDH/G6P/NADP^+^ ternary complex together with EA was checked by means of molecular dynamics (MD) simulation, previously validated on the 4EM5 complex. Analysis of the top-ten docking poses (Table S1) shows that the inhibitor occupies a cavity at the G6P-binding pocket that extends to the catalytic site and is sandwiched between G6P and residues D^79^LAKKK^84^ from helix α1 (Figure 2 and Figure S4).

In the most represented (five out of 10) poses (see a representative pose in Figure 2), EA shows the following conformation and interactions: (i) the 10β- and 13β-methyl groups face helix α1; (ii) the 3β-hydroxyl group is oriented outside the catalytic site, pointing towards a positively charged region formed by K84 and R408, residues that participate in binding the phosphate group of G6P; (iii) the oxygen of the 3β-OH group is at a distance for H-bonds with the NH_2_ from the side chain of R408 and K84, whereas the H from the 3β-OH group may establish weak polar interactions with the phosphate of G6P; (iv) the 5-membered D-ring of EA is placed close to the nicotinamide, where the non-polar side chain of L80 provides an hydrophobic environment to stabilize it. Furthermore, electrostatic interaction between K83 and D79 contributes to stabilize the N-terminal region of helix α1 and the position of L80.

It is worth noting, the docking model shows that EA does not affect substrates binding but instead interferes with catalysis by positioning them into non-catalytically competent orientations. In this respect, the binding of nicotinamide to G6PDH is destabilized by the 5-membered d-ring of EA that hinders the interaction between both substrates. In addition, the planar and rigid conformation of the EA ring system and its angular methyl groups, which intercalate between structural elements and residues important for catalysis, provide additional structural restraints in the catalytic site of the protein. In summary, this binding model of EA to *Tc*G6PDH is compatible with the uncompetitive inhibition mechanism reported for steroids.

### 2.2. Biochemical Validation of EA Binding to TcG6PDH

To further validate the *in silico* model of the holo-*Tc*G6PDH-EA complex, point mutations of residues key for stabilizing steroid binding were generated and kinetically characterized (Table 1).

As pointed out above, L80 is at the N-terminus of a helix that contains residues key for G6P-binding, and provides a hydrophobic environment suitable for binding the nicotinamide ring of NADP^+^ and the 5-membered D-ring of EA. This residue was replaced by a glycine, which presents a significantly less bulky and hydrophobic side chain. As expected, the L80G mutation did not affect the apparent K_M_ for G6P (K_M_ = 74 µM) but increased 4.7-fold the apparent K_M_ for NADP^+^ (K_M_ for NADP^+^ = 75 µM) when compared to the parameters exhibited by the WT enzyme (K_M_ for G6P = 77 µM and K_M_ for NADP^+^ = 16 µM). Interestingly, the *kcat* value of the L80G mutant decreased by 20 to 30-fold (2.7–2.0 s^−1^) with respect to that of WT *Tc*G6PDH (*kcat* ~ 52–62 s^−1^), suggesting that this residue plays an important role during catalysis. In agreement with the changes observed in the kinetic parameters and the docking model, the inhibition constant (Ki) of the mutant L80G for EA was one order of magnitude higher (Ki EA = 31 µM) than that corresponding to WT *Tc*G6PDH (Ki EA = 2.5 µM), indicating a significant destabilization of inhibitor binding in the absence of Leu.

According to the docking model, the side chains of the lysine residues at position 83 and 84 are engaged in polar interactions with D79 and the 3β-OH of EA, respectively (Figure 2). In addition, the model of the (catalytic) ternary complex shows that both residues interact with the phosphate group of G6P (not shown). In support of the *in silico* models, the replacement of K83 or K84 by alanine produced an almost 7- to 8-fold increase in the apparent K_M_ for G6P (K_M_ for G6P = 537 µM and 618 µM for K83A and K84A, respectively) and, a negligible change in the apparent K_M_ for NADP^+^ (K_M_ for NADP^+^ = 21 and 17 µM, for K83A and K84A, respectively), with respect to the reference parameters of the wildtype enzyme (K_M_ for G6P = 77 µM and K_M_ for NADP^+^ = 16 µM; Table 1). Similarly to the mutant L80G, replacement of K83 entailed a marked decrease (~20-fold) in the *kcat* that affected negatively enzyme turnover with both substrates and rendered *kcat*/K_M_ values 20 to 160-fold lower than those corresponding to the WT enzyme (Table 1). In contrast, catalysis was not significantly affected in the mutant K84A, where the 2-fold lower *kcat* obtained at varying concentrations of G6P is explained by the increased apparent K_M_ (618 µM) for this substrate and the not fully saturating concentration (5 mM G6P) used during the kinetic characterization of the mutant. As described above, the docking model of EA shows that both residues are directly (K84) or indirectly (K83) involved in EA-binding to *Tc*G6PDH, which was confirmed by the 3- to 4-fold increase in the Ki for EA determined for K83A (Ki = 11 µM) and K84A (Ki = 8 µM), respectively. Taking together, the biochemical and structural data led us to propose that K83 fulfils an important structural role by providing helix 1 with the right orientation to bind G6P (in a catalytically competent mode) or EA and demonstrate that K84 is engaged in G6P- and EA-binding.

As mentioned in the previous section, the 3β-OH group of EA is also at distance to establish an H-bond with the side chain of R408 and contributes to G6P binding during catalysis. In addition, the catalytic model shows that R408 is not exclusively involved in G6P binding but also engaged in a polar contact with a negatively charged residue, namely E285, from a loop close to the catalytic site (Figure S1). In order to determine the real contribution of R408 to inhibitor binding, this residue was exchanged by an alanine. Compared to the WT enzyme, the R408A mutant displayed an almost identical apparent K_M_ for NADP^+^ (17 μM), a ~2-fold higher K_M_ for G6P (168 μM) and a >3-fold decrease in the *kcat*. Thus, the kinetic behavior of this mutant is fully compatible with the stabilizing role assigned to R408 in G6P-binding during catalysis. Because the Ki for EA of the mutant R408A was almost identical (Ki EA = 3 μM) to that of the WT enzyme, it can be concluded that R408 is not a major determinant for steroid binding.

### 2.3. Activity and Binding Mode to TcG6PDH of Novel EA Derivatives

**Figure 3:**
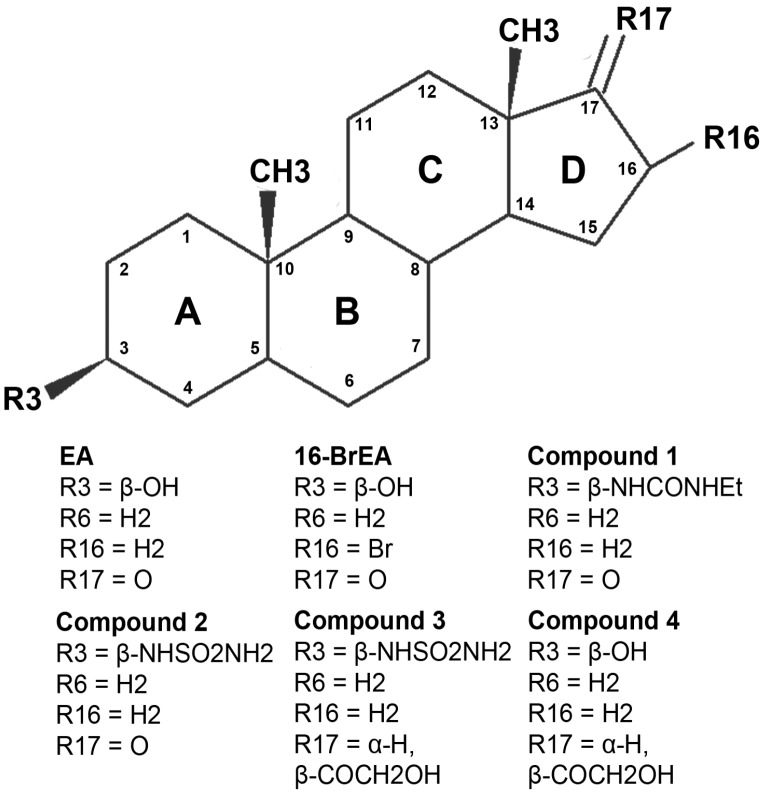
Chemical structure of epiandrosterone (EA) and derivatives analyzed in this study. EA, epiandrosterone; 16-BrEA, 16-bromo epiandrosterone; **1**, 3β-ethylurea analogue of EA; **2**, 3β-sulfamide analogue of EA; **3**, 21-hydroxypregnan-20-one analogue of **2**; **4**, 21-hydroxypregnan-20-one analogue of EA.

The activity and binding mode of a series of EA derivatives (Figure 3) to *Tc*G6PDH were studied. The compounds were selected for containing substitutions in positions that, according to the docking model for EA, are key for steroid binding and *Tc*G6PDH inhibition.

The EA analogues subjected to biochemical (Table 2) and/or *in silico* analysis (Table S1) are: 16-bromo epiandrosterone (16-BrEA), a derivative with a 3β-ethylurea (compound **1**) or a 3β-sulfamide (compound **2**), and a 21-hydroxypregan-20-one with a 3β-sulfamide (compound **3**) or a 3β-alcohol (compound **4**).

**Figure 4:**
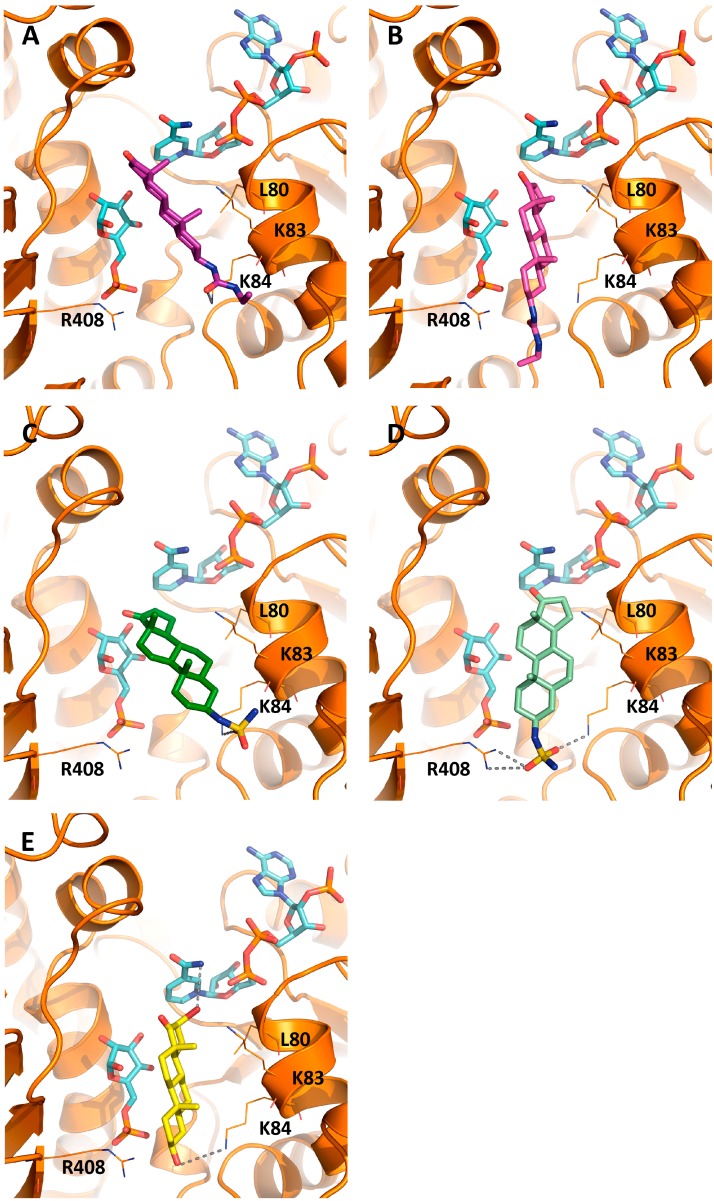
Binding modes of 3β- and 16β-substituted androstanes to the catalytic complex of *T. cruzi* G6PDH. *Tc*G6PDH structure and residues relevant for inhibitor binding are shown in orange cartoon and lines. The substrates are depicted in sky blue sticks and the H-bonds with grey dashed lines. The figures show the most stable pose for (**A**) **1** (dark purple sticks) and (**D**) **2** (pale green sticks) and alternative binding conformations for (**B**) **1** (purple sticks) and (**C**) **2** (dark green); (**E**) Best docking pose for 16Br-EA, showing the 5-membered d-ring group occupying the active site and the bromide atom establishing a halogen bond with the amide of NADP^+^.

Overall, docking of steroids **1** and **2** to the *Tc*G6PDH ternary complex shows that the corresponding 3β-substituents determine two possible orientations of the androstane moiety in the binding site. Binding of **1** to helix α1 is dictated by H-bonds between the 3β-urea and the NHε of K84 (Figure 4A, Figure S5 image A). This binding conformation together with the rigid structure of the androstane determines that the 5-membered D-ring protrudes towards the space between both substrates at the catalytic site (Figure 4A, Figure S5A). In contrast, an H-bond between the 3β-urea oxygen and the side chain of R408 favors binding of **1** parallel to the substrate and with its D-ring occupying only a minor volume of the catalytic pocket (Figure 4B, Figure S5B).

Binding of compound **2** to *Tc*G6PDH occurs in the same pocket used by EA and **1**. In several conformers, the inhibitor is anchored to helix α1 through an H-bond between one sulfamide oxygen and the NHδ of K84 (Figure 4C and Figure S5C). In an alternative and more stable binding conformation (ΔG ≈ −14.37 kcal/mol), the sulfamide moiety of the inhibitor establishes H-bonds with the side chains of K84 and R408 (Figure 4D and Figure S5D). Independently of the conformation adopted by compound **2**, the angular methyl groups point towards the solvent and the 17-ketone group, although located slightly further from the active site, is able to provide steric hindrance at the catalytic site.

Compared to the parental scaffold, the inclusion of polar substitutions at position 3β of EA (compound **1** and **2**) increased by ~10-fold the inhibition of human G6PDH [16] but only moderately (≤2-fold) their potency towards the parasite enzyme (Table 2). This suggests a species-specific binding mode of steroids to G6PDH.

If the binding models obtained for EA and the 3β derivatives **1** and **2** are correct, then the addition of bulky groups at position 16 or 17 may be beneficial or detrimental for inhibition depending on the nature and size of the substituent. To test this, derivatives of EA with a bromide atom at position 16 (16-Br EA) or a 21-hydroxypregan-20-one group at position 17 with a 3β-sulfamide (compound **3**) or a 3β-alcohol (compound **4**) were assayed against the recombinant enzyme and/or their potential binding modes simulated *in silico*.

In 7 out of 10 docking poses, the binding mode of 16α-Br EA to *Tc*G6PDH resembled that of EA. In the most stable protein-ligand complex (ΔG −12.07 kcal/mol), the steroid makes a full occupancy of the catalytic site and fixes the substrates in conformations not suitable for catalysis (Figure 4E and Figure S5E). The major interactions between the enzyme-substrate complex and the inhibitor are: an H-bond between the 3β-OH and the NHδ of K84 and an halogen bond between the bromide and the hydrogen from the terminal amide of nicotinamide (Figure 4E). Although weaker in strength with respect to hydrogen bonds, this Br···H-N bond contributes to increase the affinity of the inhibitor for bound NADP^+^ while restraining the mobility of the nicotinamide ring to approach G6P. This likely explains the 65- and 115-fold higher potency of the 16-halogenated congeners of EA and DHEA, respectively, towards *Tc*G6PDH [10] (Table 2). Notably, this derivative is almost two orders of magnitude less active against the human G6PDH [12,16,24].

**Figure 5:**
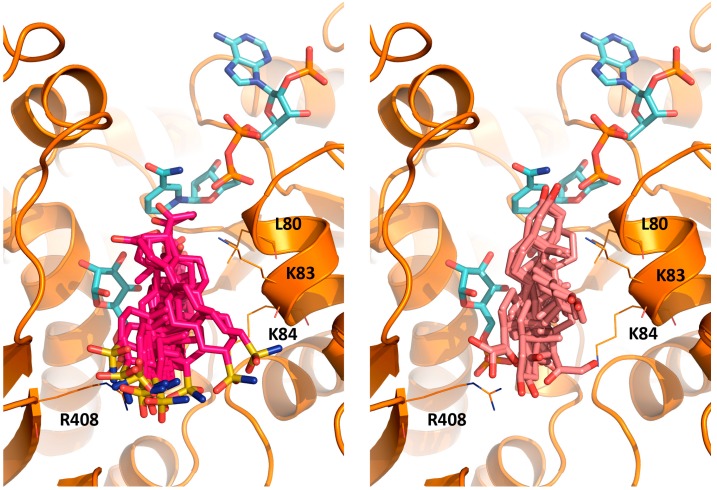
Binding of **3** and **4** to *Tc*G6PDH in complex with G6P and NADP^+^. *Tc*G6PDH structure is shown as orange cartoon and residues participating in interactions are shown in lines with substrates depicted in sky blue sticks. The different poses obtained during the docking studies are shown for **3** (dark pink sticks) and **4** (pink sticks).

Molecular docking of steroids **3** and **4** shows that both compounds occupy the steroid binding site of *Tc*G6PDH with orientations determined by polar interactions of the 3β-sulfamide (**3**) and 21-hydroxypregnan-20-one (**4**) groups with K84 and R408 (Figure 5).

However, the occupancy of the cavity by **3** and **4** is not full and, for all poses, the d-ring localizes distant from the catalytic site, which agrees with their lower inhibitory activity (60%–70% inhibition at 30 μM compound; Table 2). This behavior contrasted with the enhanced anti-human G6PDH activity reported for these compounds (IC_50_ ~ 2 μM) but is not surprising since compounds reported to target *Tc*G6PDH do not inhibit well the human enzyme [16].

**Figure 6:**
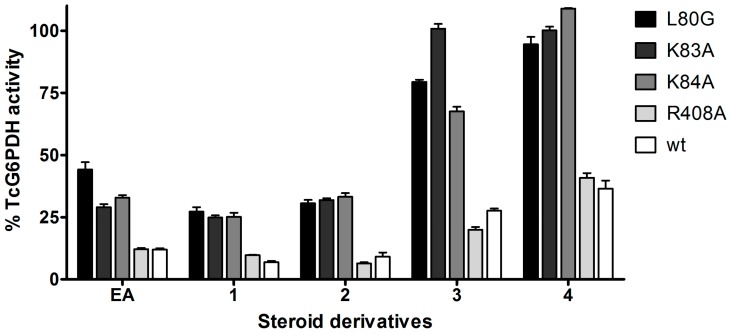
Inhibitory activity of steroids toward *Tc*G6PDH WT and mutants. The inhibitory activity of EA and derivatives thereof was tested against the wildtype and mutants L80G, K83A, K84A or R408A of *Tc*G6PDH, as described in Section 3.4. of the Experimental Section. The activity is shown as a percentage relative to the corresponding non-treated enzyme controls and compounds were tested at a fixed concentration of 30 µM.

In order to confirm the binding preference of steroids suggested by the docking models, the activity of EA analogues was tested towards *Tc*G6PDH mutants using saturating concentrations of substrates and compounds at a final concentration of 30 µM (Figure 6). As previously observed for EA (Table 1), only mutations in L80, K83 and K84 but not in R408 affected significantly and negatively the inhibitory activity of compounds **1** to **4** (Figure 6), pointing to an important stabilizing effect of these residues in steroid binding as suggested by the docking models (Figure 2 and Figure 4).

A recent *in silico* study proposes a binding region for steroids in human G6PDH [14] that differs from the one elucidated here for the *T. cruzi* enzyme. Although this may explain the species-specificity observed for certain steroids, such an assumption must be taken with care because the docking models obtained for the human enzyme show a partial occupancy of the nicotinamide binding site by the inhibitors, which is not compatible with the uncompetitive inhibition mechanism exerted by steroids. This inconsistency probably stems from the use of the binary (human G6PDH-G6P) and not the ternary enzyme-substrate complex as a template for the dockings.

## 3. Experimental Section

### 3.1. Plasmids

The expression vector for N-terminally His-tagged full-length (pET28a (+)-*Tc*G6PDH_L_; Accession Nr. ABD72517.1) was a kind gift of Juan José Cazzulo (Universidad de San Martín, CONICET, Buenos Aires, Argentina) [7]. The single Lys84Ala, Leu80Gly, Arg408Ala and Arg408Lys mutants of *Tc*G6PDH were generated with the Quick Change site-directed mutagenesis kit (Stratagene, La Jolla, CA, USA) using the parental plasmid as template and the primer pairs K83A Fwd 5′ CTCGGTGCAAGCGGGGACTTGGCCAAAGCGAAGACCTTTCCGGCG 3′/K83A Rev 5′ CGCCGGAAAGGTCTTCGCTTTGGCCAAGTCCCCGCTTGCACCGAG 3′ K84A Fwd 5′ GGGGACTTGGCCAAAAAGGCGACCTTTCCGGCGCTTTTT 3′/K84A Rev 5′ AAAAAGCGCCGGAAAGGTCGCCTTTTTGGCCAAGTCCCC 3′, and L80G Fwd 5′ CTCGGTGCAAGCGGGGACTCCGCCAAAAAGAAGACC 3′/L80G Rev 5′ GGTCTT CTTTTTGGCGGAGTCCCCGCTTGCACCGAG 3′, R408A Fwd 5′ CCGGAAAGGCACTC GAAGAGGCTCTGCTTGATATCCGTATTCAGTTCAAGGAC 3′/R408A Rev 5′ GTCCT TGAACTGAATACGGATATCAAGCAGAGCCTCTTCGAGTGCCTTTCCGGC 3′ (underlined are indicated the mutated bases), respectively. The PCR were performed in a total reaction volume of 50 μL according to the instructions of the supplier. One Shot^®^ MAX Efficiency^®^ DH5α™-T1R cells were transformed with the mutagenesis reaction. The correctness of the targeted mutations was confirmed in at least three independent plasmids from each construct by DNA sequencing (Molecular Biology Unit, Institut Pasteur de Montevideo, Montevideo, Uruguay).

### 3.2. Expression and Purification of Recombinant Proteins

The wild-type (WT) form and mutants (K83A, K84A, L80G and R408A) of *Tc*G6PDH_L_ were expressed in *Escherichia coli* BL21 (DE3) grown in ZYM-5052 auto-induction medium. Briefly, an overnight culture of transformed bacteria grown in LB was inoculated at a ratio 1:100 in ZYM-5052 containing 50 µg/L kanamycin, and incubated for 48 h at 25 °C and 200 rpm. Cells were harvested by centrifugation (5000× *g*, 10 min, 4 °C) and resuspended in 50 mM Tris, pH 8.0, 500 mM NaCl, 5 mM MgCl_2_ (buffer A) containing 1 mM PMSF, 40 µg/mL TLCK, 150 nM pepstatin, 4 nM cystatin and 1 mg/mL lysozyme. After incubating for 45 min at 4 °C, the cell lysate was subjected to three cycles of sonication (30 pulses per minute at 55% amplitude with a macrotip) in a Digital Sonifier 450 (Branson, Danbury, CT, USA) and then cleared by centrifugation at 27,000× *g* (45 min, 4 °C) followed by filtration in a 0.45 µm nitrocellulose filter (Millipore, Billerica, MA, USA). The cleared lysate was loaded onto a HisTrap column (GE-HealthCare, Little Chalfont, UK) pre-equilibrated with buffer A and unbound proteins were removed with 5 column volumes of buffer A. After washing the column with buffer A containing 5 mM imidazole, the His-tagged proteins were eluted with 500 mM imidazole in buffer A and concentrated by diafiltration (Ultra-15 30K NMWL filter, 4 °C, 7000× *g* Amicon, Darmstadt, Germany). The final purification was performed in an AKTA Purifier system, using a Superdex 200 10/300 GL column (GE-HealthCare) pre-equilibrated with buffer A. Enzyme purity and concentration was assessed by Coomasie blue stained SDS-PAGE gels and the bicinchoninic acid assay (Bicinchoninic Acid Protein Assay Kit, Sigma, St. Louis, MO, USA), respectively. G6PDH activity was determined as described below. The protein was concentrated to 0.5 mg/mL and stored at 4 °C.

### 3.3. Kinetic Assays

G6PDH activity was determined at ~25 °C by monitoring NADPH (ε340 = 6220 M^−1^·cm^−1^) formation at 340 nm. All assays were performed in buffer B (50 mM Tris pH 7.5, 5 mM MgCl_2_) in a final reaction volume of 150 µL and started by addition of glucose-6-phosphate (G6P). For WT *Tc*G6PDH, the Michaelis’ constants (K_M_) and the maximum initial velocity (Vmax) were determined varying reciprocally the substrate (10, 50, 200, 1000 and 5000 µM G6P) and coenzyme (5, 10, 50, 250 and 625 µM NADP^+^) concentrations. For the mutants, the kinetic characterization was performed at a fixed saturating concentration of the corresponding co-substrate (*i.e.*, 6 mM G6P and 700 µM NADP^+^) and varying concentrations of the substrate (25, 50, 70, 100, 250, 350, 500 and 700 µM NADP^+^, and 0.005, 0.025, 0.05, 0.25, 0.65, 1, 2.5 and 5 mM G6P). The concentration of enzyme in the assay was of 9.7, 970, 534, 316 and 100 nM for WT, K83A, K84A, L80G and R408A respectively. The first 30 s of the reaction were used to calculate initial velocities that were normalized by the corresponding enzyme concentration in the assay. All measurements were performed in triplicate using a Cary 50 Bio spectrophotometer (Agilent Technologies, Santa Clara, CA, USA). The initial rates were determined with the Origin Pro 8.0 software (OriginLab Corporation, Northampton, MA, USA), and the apparent kinetic constants were calculated by nonlinear regression using the GraphPad Prism 5.0 software (GraphPad Inc., San Diego, CA, USA). The data were fitted to the Cleland or Michaelis-Menten equation to obtain the corresponding kinetic parameters.

### 3.4. Inhibitor Assays with Wildtype or Mutants of TcG6PDH

For all inhibition assays with wildtype or mutants of *Tc*G6PDH_L_, initial reaction rates under saturating conditions of both substrates (NADP^+^ 0.7 mM and G6P 6 mM) and different concentrations of steroidal compounds were measured at 340 nm with a Cary 50 Bio spectrophotometer. Stock and working solutions of compounds were prepared in 100% (*v*/*v*) DMSO. The reactions were performed in a quartz cuvette containing 5 µL of inhibitor solution and 245 µL buffer B added of 1.4 mM NADP^+^ and 12 mM G6P and started by addition of 250 µL of an enzyme solution in Buffer B. The final concentration of enzyme in the assay was: 698 nM for *Tc*G6PDH K83A, 495 nM for *Tc*G6PDH K84A, 380 nM for *Tc*G6PDH L80G, 40 nM for *Tc*G6PDH R408A, and 48 nM (for assays with EA) or 13 nM (for assays with steroid derivatives) for *Tc*G6PDH wildtype. A reaction control with 1% (*v*/*v*) DMSO was run for each enzyme species. The activity of the steroid derivatives EA and **1** to **4** was initially assayed at 30 μM against wildtype and mutants of *Tc*G6PDH. To obtain IC_50_ values for EA and the most potent derivatives, the activity of the compounds was tested at concentrations of 0.5, 5, 10, 25, 50, 100, 250 µM, and 0.5, 1.5, 3.0, 7.5, 15, 30 µM, respectively. The IC_50_ was estimated from non-linear regression fitting of initial velocity *vs.* [compound] plot using the program GraphPad prism 5.0. The Ki was calculated with the equation [25]:

Ki = IC_50_/(K_M_/[S] + 1)

### 3.5. Generation of TcG6PDH/G6P/NADP^+^ Ternary Complex and Steroids Docking

The crystal structure of a truncated form of *Tc*G6PDH lacking the first 37 amino acids and co-crystallized in presence of its physiological substrate G6P (PDB ID: 4EM5; resolution 3.35 Å) [15] was pretreated by means of the Protein Preparation Wizard tool of the Maestro suite 9.2 [26] at default settings, adding hydrogen atoms, deleting water molecules, fill in missing amino acids side chains and eventual missing loop using Prime [27], generating ionization states of the co-crystallized ligand at pH 7 ± 3.0 using Epik [28] and checking amino acid protonation state using Propka, always at physiological pH [29]. NADP^+^, EA and all the other steroid structures discussed here were modeled with LigPrep [30] at default settings. In order to obtain a complete protein model, the missing NADP^+^ was docked in the pretreated chain C of the tetramer *Tc*G6PDH by means of induced fit docking (Maestro suite 9.2) [31]. The induced fit algorithm is designed to reproduce conformational changes in the protein binding site upon ligand binding, using both Glide and Prime. In order to generate a diverse ensemble of ligand poses, the procedure uses reduced Van der Waals radii and an increased Coulomb-vdw cutoff, and can temporarily remove highly flexible side chains during the docking step. For each pose, a Prime structure prediction is then used to accommodate the ligand by reorienting nearby side chains. These residues and the ligand are then minimized. Finally, each ligand is re-docked into its corresponding low energy protein structures and the resulting complexes are ranked according to the GlideScore. Based on the inhibition mechanism exerted by EA on *Tc*G6PDH, the ligand and the other steroid inhibitors were docked in the catalytic site of the *Tc*G6PDH/G6P/NADP^+^ complex. The docking grid was centered on G6P with an inner box size of 20 × 20 × 20 Å and an outer box size of 40 × 40 × 40 Å. Docking runs were performed using the Glide Standard Precision (SP) scoring function, choosing the best 50 structures undergoing the post-docking energy minimization, saving the very best 10 poses showing a threshold energy lower than 0.50 kcal·mol^−1^. The 10 final poses were then rescored by means of SZYBKI 1.8.0.1 [23], using the Poisson-Boltzmann solvation model [32]. Model images were generated and analyzed using PyMol 1.7.1.3 [33].

### 3.6. MD Simulation of TcG6PDH/G6P/NADP^+^/EA Complex

The MD simulation of the *Tc*G6PDH/G6P/NADP^+^/EA complex has been performed by means of NAMD 2.10 [34] using the CHARMM 36 force field [35]. For the system set up, a first minimization in vacuum (only H atoms and then the whole system for a total of 2000 minimization steps) has been performed, followed by solvation of the minimized complex in a TIP3P water box 10 Å longer than the protein in all six directions. Six neutralizing Na^+^ ions were added. During production MD, temperature was kept constant at 310 K by coupling all non-hydrogen atoms to a Langevin thermostat with a friction coefficient of 5 ps^−1^. Non-bonded interactions were cut off above 10 Å and smoothed to zero beginning from 9 Å. PME long range electrostatic with a grid spacing of 2 Å was used, and all bonds involving hydrogen atoms were constrained using RATTLE [36]. Production runs have been performed at constant pressure (NPT ensemble), for a total of 80 ns with a time step of 2 fs.

## 4. Conclusions

Our study discloses the putative site binding of steroids to the ternary complex of *Tc*G6PDH, which involves a groove that runs alongside the G6P-binding site and helix α1 with its aliphatic (K83 and K84) and hydrophobic (L80) residues that provide stabilizing interactions. Such a binding mode of EA does not affect substrate binding but directly interferes with catalysis because: (i) it affects the dynamic of helix α1—a structural element that shapes the catalytic site and participates in NADP^+^ and G6P binding; (ii) provides steric hindrance for the interaction of the nicotinamide group of NADP^+^ with G6P via its d-ring group that protrudes into the catalytic site; and (iii) delays the exit of G6P or, eventually, its product (6-phosphogluconolactone) from the active site.

Furthermore, SAR analysis of EA analogues supported by docking models provides clues towards the determinants for the selective inhibition of the pathogen enzyme by steroids: (i) polar substitutions at position 3β of EA were well tolerated and rendered congeners that increased moderately the activity against *Tc*G6PDH; (ii) a bromide atom at position 16 of EA favors the formation of an halogen bond with NADP^+^, which increases the affinity for the inhibitor and the steric effect on the catalytic site of *Tc*G6PDH; (iii) in contrast to the human enzyme, polarizable groups at position 17β of EA or compound **1** have a destabilizing effect on inhibitor binding to *Tc*G6PDH, with the steroids exploring different binding conformations that are less suited to interfere with catalysis. Taking together, these data also suggest the presence of different binding orientation or sites for steroids in the host and pathogen enzyme.

The structural information obtained from this study may prove valuable to investigate the binding mode of the recently discovered new uncompetitive inhibitors of *T. cruzi* G6PDH [12] as well as for the challenging design of more potent and selective inhibitors targeting the pathogen enzyme.

## Figures and Tables

**Table 1:** Kinetic parameters of wildtype and point mutants of recombinant *Tc*G6PDH with substrates ^a^ and the inhibitor epiandrosterone (EA).

G6PDH	K_M_ G6P (µM)	*kcat* (s^−1^)	*kcat*/K_M_ G6P (s^−1^·µM^−1^)	K_M_ NADP^+^ (µM)	*kcat* (s^−1^)	*kcat*/K_M_ NADP^+^ (s^−1^·µM^−1^)	K_i_ EA ^b^ (µM)
WT	77 ± 20	62 ± 3	0.8	16 ± 3	52 ± 2	3.2	2.5 ^c^
L80G	74 ± 17	2.0 ± 0.1	0.027	75 ± 9	2.7 ± 0.1	0.036	31 ^d^
K83A	537 ± 99	2.8 ± 0.2	0.005	21 ± 3	3.0 ± 0.1	0.14	11 ^e^
K84A	618 ± 78	24.6 ± 0.9	0.04	17 ± 4	58 ± 2	3.4	8 ^f^
R408A	168 ± 7	15.0 ± 0.3	0.089	17 ± 3	19 ± 1	1.1	3 ^g^

^a^ The apparent kinetic parameters were determined from non-linear regression plots of velocity *vs.* [substrate] from triplicate experimental determinations; ^b^ The Ki for EA was calculated using the equation: Ki = IC_50_/(K_M_/[S] + 1); If S >> K_M_, Ki ≈ IC_50_ and the corresponding associated errors for each mutant are: ^c^ 12%; ^d^ 3.2%; ^e^ 22%; ^f^ 13% and ^g^ 21%.

**Table 2:** Inhibition of *Tc*G6PDH by steroids.

Compound	Substituent				IC_50_ (µM) ^a^	G6PDH Activity [%] ± SD at 30 µM Compound ^b^
	3-α	3-β	17-	16-α		
EA	H	OH	O	H	3.0 ± 0.4 ^c^	12 ± 2
16Br-EA	H	OH	O	Br	0.015 (13.3 to 16.6) ^d^	ND ^e^
**1**	H	NHCONHEt	O	H	1.5 (1.0 to 2.0) ^f^	7 ± 1
**2**	H	NHSO_2_NH_2_	O	H	2.2 (1.8 to 2.9) ^f^	9 ± 1
**3**	H	NHSO_2_NH_2_	α-H, β-COCH_2_OH	H	ND ^e^	30 ± 2
**4**	H	OH	α-H, β-COCH_2_OH	H	ND ^e^	43 ± 2

^a^ The IC_50_ was determined from dose/response curves with 7 point concentrations tested in triplicate; ^b^ The values are expressed as the mean % G6PDH activity with respect to the control without inhibitor from triplicate experimental determinations; ^c^ The value is expressed as mean ± standard deviation; ^d^ Value reported by [12] with confidence interval provided within brackets; ^e^ ND, not determined; ^f^ IC_50_ values with corresponding confidence intervals.

## Supplementary Materials

Supplementary materials can be accessed at: http://www.mdpi.com/1420-3049/21/3/368/s1.
