# Supplementary material for: Binding Mode and Selectivity of Steroids towards Glucose-6-phosphate Dehydrogenase from the Pathogen Trypanosoma cruzi
Source: Molecules. 2016 Mar 17;21(3):368. doi: 10.3390/molecules21030368 (PMC6273692; doi:10.3390/molecules21030368)
Supplement: Supplementary file 1 [file molecules-21-00368-s001.pdf]

Supplementary Materials: Binding Mode and Selectivity of Steroids towards Glucose-6-Phosphate Dehydrogenase from the Pathogen *Trypanosoma cruzi*

Cecilia Ortiz, Francesca Moraca, Andrea Medeiros, Maurizio Botta, Niall Hamilton and Marcelo A. Comini

Table S1. Binding ΔG values (kcal/mol) for different binding poses obtained for the respective inhibitors on the energy minimized ternary complex of *Tc*G6PDH. In bold are highlighted the poses for the corresponding compounds shown in Figures 2, 4 and 5.

	ΔG (kcal/mol)					
**Docking Pose**	**EA**	**16Br-EA** [a]	**1**	**2**	**3**	**4**
1	**−11.458**	−6.509	−11.717	**−11.096**	**−11.325**	−10.595
2	−9.824	−10.750	−10.236	−7.661	−9.467	−12.191
3	−6.471	0.480	**−13.307**	−9.205	−6.108	−11.690
4	−7.420	**−12.069**	**−10.058**	**−14.368**	−11.700	**−11.344**
5	−7.670	−9.013	−9.641	−9.727	−8.224	−5.725
6	−7.767	−8.954	−3.826	−10.398	−9.268	**−14.421**
7	−8.476	−10.423	−8.357	−7.805	−9.980	−11.010
8	−11.323	−0.652	−2.281	−8.161	**−13.918**	−4.379
9	−10.838	−-3.979	−6.088	−4.704	−9.925	−5.400
10	−9.768	−6.391	−9.500	−4.586	−11.675	−10.757

[a] For 16Br-EA was investigated the R-enantiomer.

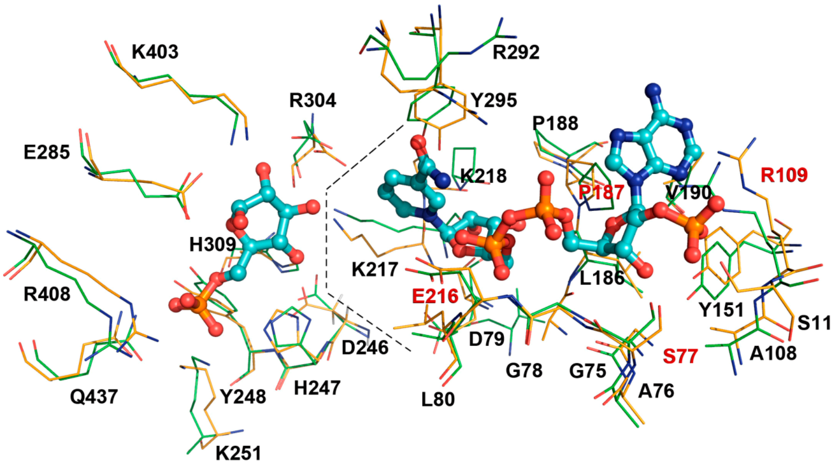

Figure S1. Comparison of the substrate binding sites for the crystal structure of *T. cruzi* G6PDH with bound G6P and the corresponding molecular model of the catalytic complex. The residues involved in substrate binding are shown for *Tc*G6PDH with bound G6P (PDB 4EM5, green lines) and for the model of the ternary complex enzyme-G6P-NADP$^+$ (bright orange lines). The dashed line denotes the separation between the N- (right side) and C-terminal (left side) domain of G6PDH. Residues from PDB 4EM5 interfering with NADP$^+$ binding are labeled with red fonts (S77, R109, P187 and E216): the OH group from S77 (β1-loop-α1) clashes with an oxygen from the phosphate bound at C2′ in the nucleotide-adenine moiety, the side chain of R109 (β2-loop-α3) clashes with the adenine ring from NADP$^+$, P187 (β4-loop-α6) clashes with the adenine ring, and the CO from E216 (β5-loop-α7) clashes with the OH group from the C2′ of NADP$^+$. During MD studies, these residues underwent conformational changes that allowed binding of NADP$^+$.

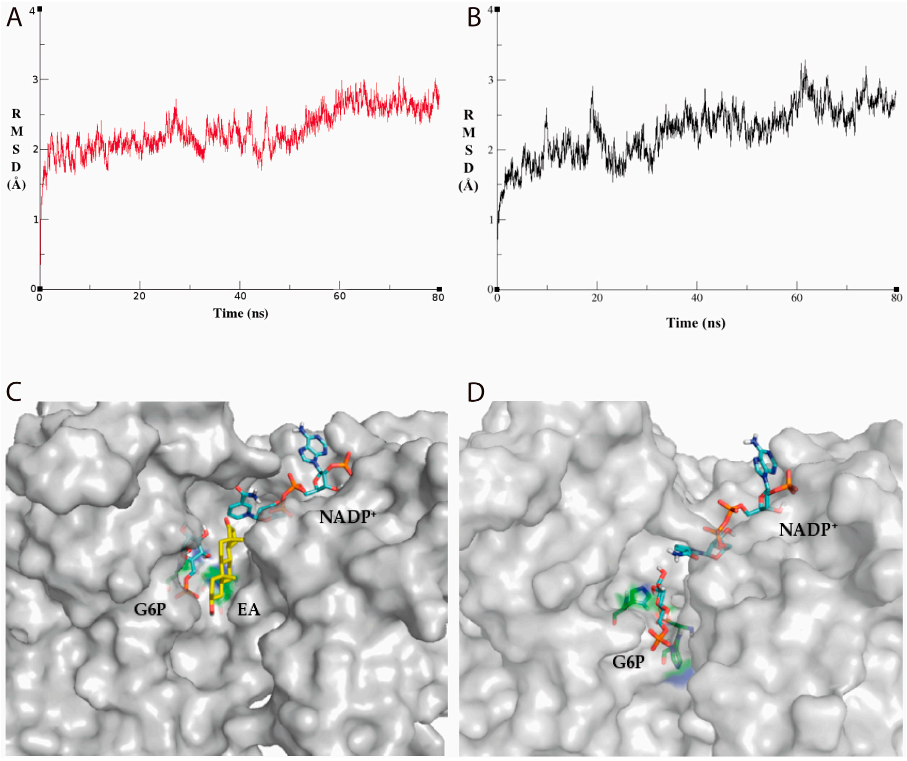

Figure S2. Molecular dynamics analysis of *Tc*G6PDH in complex with ligands. Root mean square deviation plot over 80 ns MD simulation for (**A**) the protein-G6P complex and (**B**) the protein-G6P-NADP^{+}-EA complex, where the deviation from the equilibrium observed after 50 ns correspond to the exit of EA from its binding site; (**C**) EA docking binding pose (yellow stick) in the ternary complex *Tc*G6PDH/G6P/NADP^{+} (protein surface is depicted in gray and substrates with cyan sticks); (**D**) Conformational change of the docking complex after 80 ns MD simulation, where EA exits the complex and G6P adopts an orientation not favorable for electron exchange with NADP^{+}.

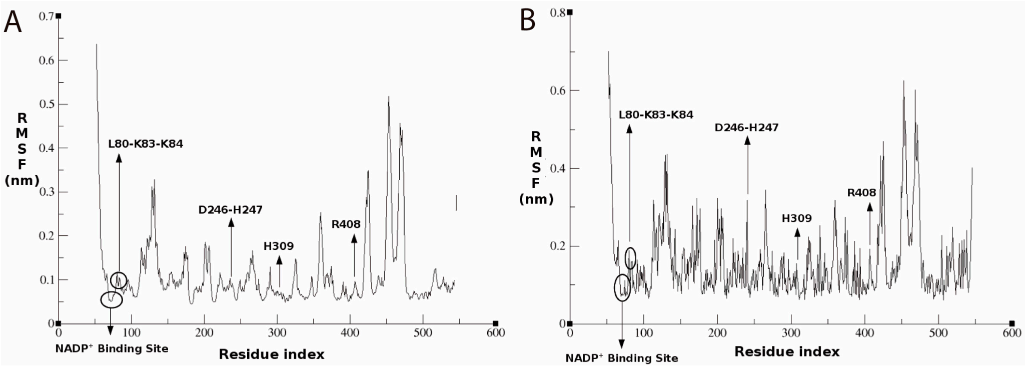

Figure S3. RMSF plots for backbone and side chains of the *Tc*G6PDH/G6P/NADP^{+}/EA complex. The computed root mean square fluctuation plots were obtained for the protein backbone (**A**) and side chains (**B**) of the holo-*Tc*G6PDH with bound EA. The high peaks (RMSF >0.1 nm for plot A or >0.17 nm for plot B) between residues 110 to 140 and residues 420 to 470 correspond to (loop rich) protein regions not engaged in ligand-binding but exposed to the solvent and participating in subunit interactions, respectively. The fluctuations encompassing the last region will be smoothed if running a simulation of the protein tetramer rather than of a single subunit (in our case chain C), but they are not expected to affect the final results since it is located far enough from the substrates- and inhibitor-binding sites. In the plots are highlighted the positions of the main residues responsible for EA binding and/or involved in catalysis (for details see text).

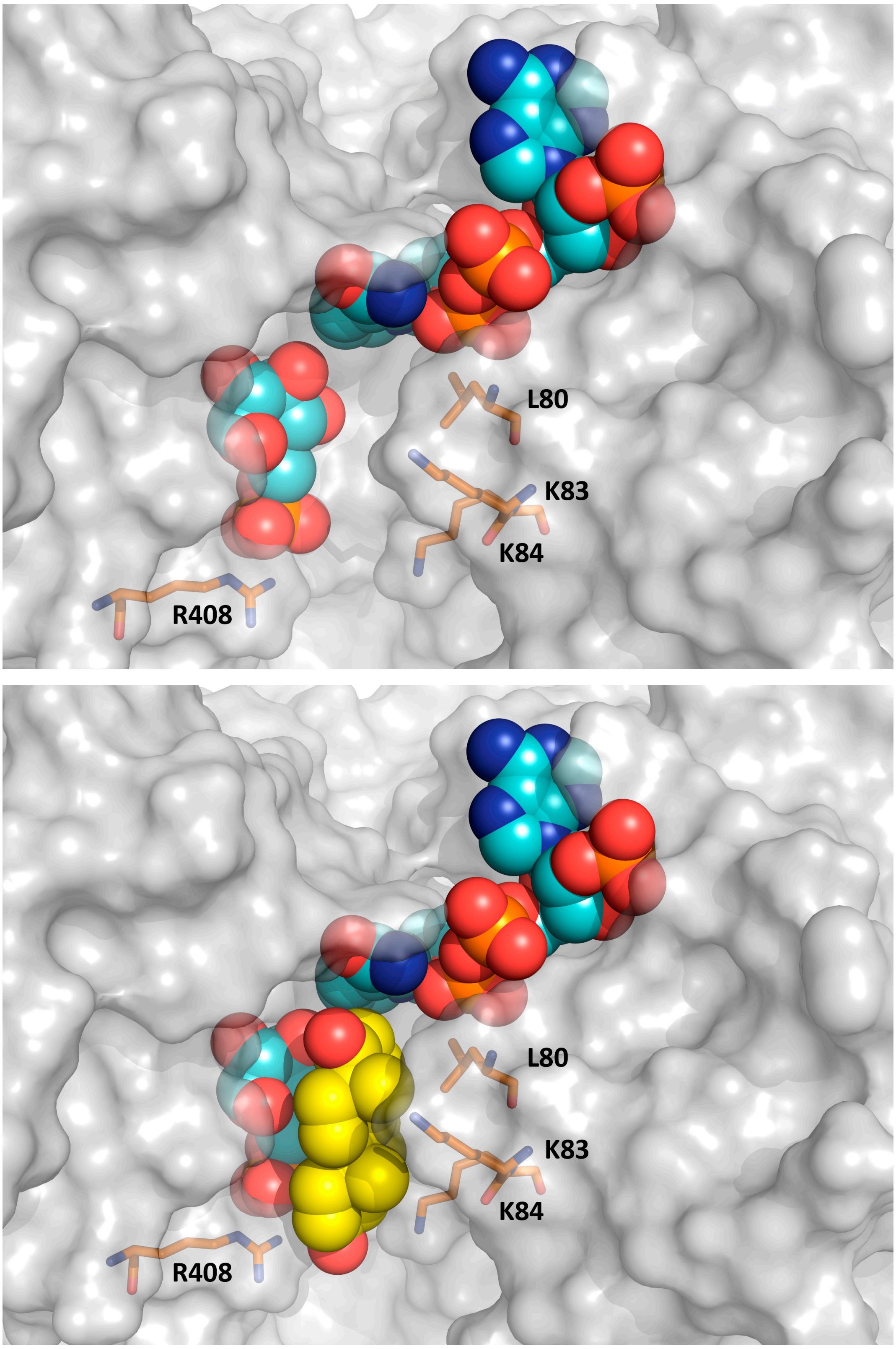

Figure S4. Binding pocket for EA in *Tc*G6PDH. Molecular model of the corresponding energy minimized quaternary complex of the enzyme (grey surface) with residues shown as bright orange sticks and substrates depicted as cyan spheres. Model without (**A**) and with docked EA (**B**), which is shown with yellow spheres.

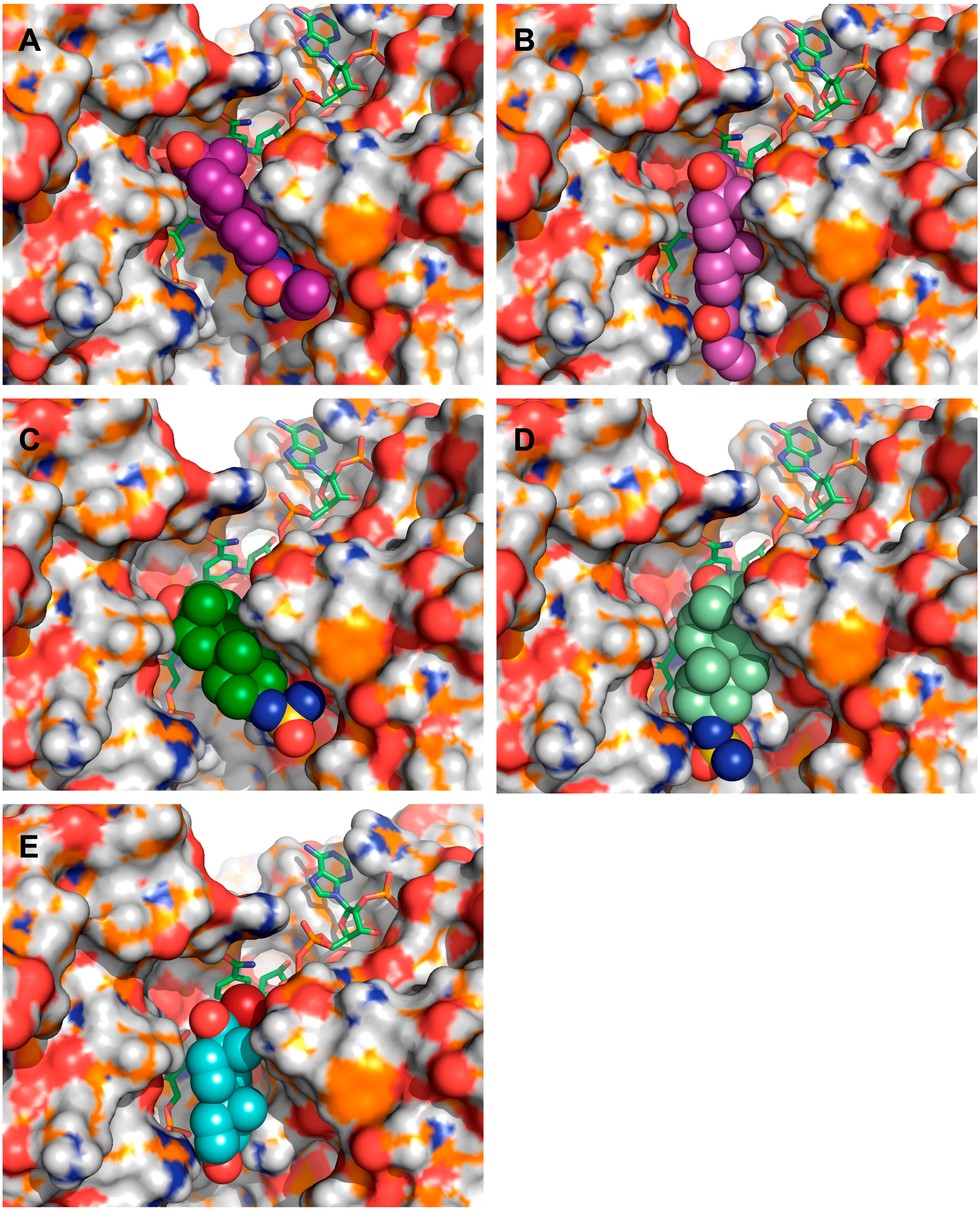

Figure S5. Binding of 3β-, 16β- and 17β-substituted androstanes to the catalytic complex of *T. cruzi* G6PDH. This figure is a replica of Figure 4 with *Tc*G6PDH shown as surface, the substrates as light green sticks and the compounds (lacking hydrogen atoms) as colored spheres. The most stable and alternative poses are shown for (**A**) **1** (dark purple) and (**D**) **2** (pale green) and alternative binding conformations for (**B**) **1** (pale purple) and (**C**) **2** (dark green); (**E**) Best docking pose for 16Br-EA, shown as light blue sphere.
